# E3 ubiquitin ligase RBX1 drives the metastasis of triple negative breast cancer through a FBXO45-TWIST1-dependent degradation mechanism

**DOI:** 10.18632/aging.204163

**Published:** 2022-07-08

**Authors:** Jun Shao, Qian Feng, Weifan Jiang, Yuting Yang, Zhiqiang Liu, Liang Li, Wenlong Yang, Yufeng Zou

**Affiliations:** 1 Department of General Surgery, The Second Affiliated Hospital of Nanchang University, Nanchang 330006, Jiangxi Province, China; 2 Department of Breast Surgery, The Third Hospital of Nanchang, Jiangxi Provincial-Key-Laboratory for Breast Diseases, Nanchang 330006, Jiangxi Province, China; 3 Department of Infectious Diseases, The Second Affiliated Hospital of Nanchang University, Nanchang 330006, Jiangxi Province, China; 4 Department of Urology Surgery, The Second Affiliated Hospital of Nanchang University, Nanchang 330006, Jiangxi Province, China; 5 Department of Medical College, Nanchang University, Nanchang 330006, Jiangxi Province, China; 6 Department of Neurology, Jiangxi Provincial People’s Hospital, The First Affiliated Hospital of Nanchang Medical College, Nanchang 330006, Jiangxi Province, China; 7 Emergency Department, Jiangxi Maternal and Child Health Hospital, Nanchang 330006, Jiangxi Province, China

**Keywords:** triple-negative breast cancer, RBX1, FBXO45, EMT, TWIST1

## Abstract

Triple-negative breast cancer (TNBC) patients are at high risk of recurrence and metastasis in the early stages, although receiving standard treatment. However, the underlying mechanism of TNBC remains unclear. Here, the critical effect of E3 ubiquitin ligase RBX1 in the metastasis of TNBC was reported for the first time. We discovered that RBX1 expression was evidently raised in the tissues of TNBC. Our clinical research displayed that high RBX1 expression was markedly related to poor distant invasion and survival. Functional analysis exhibited that RBX1 facilitated metastasis of TNBC cells through increasing EMT. Furthermore, we demonstrated that RBX1 knockdown increased the levels of the Twist family bHLH transcription factor 1 (TWIST1), is a significant regulator in the EMT process in some cancers. It can be observed an evident positive correlation between the TWIST1 and RBX1 levels, further confirming that EMT induced by RBX1 in TNBC cells is determined by TWIST1. Mechanistically, RBX1 modulates the expression of TWIST1 via modulating FBXO45, directly binding to FBXO45, and facilitating its degradation and ubiquitination. Briefly, our findings confirm that RBX1 is probably a new biomarker of TNBC carcinogenesis, thus suggesting that targeting the RBX1/FBXO45/TWIST1 axis may be an underlying strategy for TNBC treatment.

## INTRODUCTION

TNBC is a highly aggressive subtype in the breast cancer [1]. At present, the lack of effective treatments, primarily on account of their genetic complexity, restricts the “targeted” therapies development. The TNBC treatment is still the greatest challenge in the treatment of breast cancer due to the lack of universally accepted chemotherapy guidelines for metastatic, adjuvant, and neoadjuvant disease [2, 3]. The fundamental biological characteristics of TNBC have been understood in detail, which is essential for developing the successful targeted therapies. Currently, a variety of researches oriented with molecular have identified some hypothetical targets of TNBC, which gives a powerful theoretical basis for developing the targeted therapies [4, 5]. However, due to the rapid metastasis of patients after treatment, targeted therapy is not satisfactory. Our current knowledge of metastatic TNBC patients is not sufficient to improve their outcomes with effective treatment.

The ubiquitin proteasome system exerts an essential effect in controlling various cellular processes and signalling pathways and modulating the protein turnover [6, 7]. The SCF complex is a multiprotein E3 ubiquitin ligase, which is composed of F-box, Cullin, Skp1proteins as well as a RING box protein (RBX1, also called ROC1) that exerts a significant effect in the ubiquitination of proteins associated with cell cycle or other proteins [8, 9]. New evidence suggests that RBX1 is a carcinogenic gene existing in several cancers, containing colon, liver, lung, ovarian, bladder, together with several other cancer cell lines [10–15]. However, the expression and clinical significance of RBX1 in TNBC are not yet clear. The exact effect of RBX1 in the TNBC progression and the underlying signalling cascades remain unclear.

Epithelial-mesenchymal transition (EMT) is the cells’ transformation from an epithelial to the mesenchymal phenotype. EMT leads to variations in cell mobility, intercellular adhesion molecules, together with cell polarity, thus causing migratory and invasive mesenchymal cells [16–18]. EMT is a process that epithelial cells isolate from parent tissue, exchange their adhesive and morphological characteristics, and next transform into the mesenchymal cells [19, 20]. This is regarded as the first stage in invasion together with metastasis. EMT is participated in the oncogenicity and drug resistance development of tumors such as breast, prostate, lung, and skin cancers [21]. TWIST1 is a basic helix-loop-helix (bHLH) transcription factor that induces EMT. TWIST1 is thought to be involved in EMT initiation in various pathological environments, involving cancer [22, 23]. TWIST1 protein is expressed in several tumours and is associated with tumour invasion and chemotherapy resistance [24]. Interestingly, we used Tandem Mass Tags (TMT)-Mass Spectrometry Proteomics analysis to determine whether knockdown of RBX1 reduced TWIST1 protein expression in TNBC cell. Nevertheless, the potential mechanisms regulating TWIST1 existing in TNBC are largely unclear. As a result, it can be hypothesized that RBX1 probably influence EMT in TNBC through modulating the expression of TWIST1.

Furthermore, it has been reflected that human TWIST1 is modulated through factors like FBXO protein 45 (FBXO45) [25]. FBXO45 modulates the genes expression whose products are participated in the gluconeogenesis [26]. Also, it is a target of degradation mediated by ubiquitin proteasome in many cells [27, 28]. Since RBX1 is the E3 ubiquitin ligase, its function is similar to that of polyubiquitin and can be used as a label targeted by the proteasome, however, it is not explicit whether RBX1 is participated in modulating the FBXO45 degradation.

The purpose of this work is to clarify the effect of RBX1 in the outcomes of patients with TNBC. We also explored the action mechanism of FBXO45 and TWIST1 in TNBC. To sum up, our data give new underlying targets for the TNBC prognosis and treatment.

## RESULTS

### RBX1 overexpression is correlated with poor outcome in patients with in TNBC

**Figure 1:**
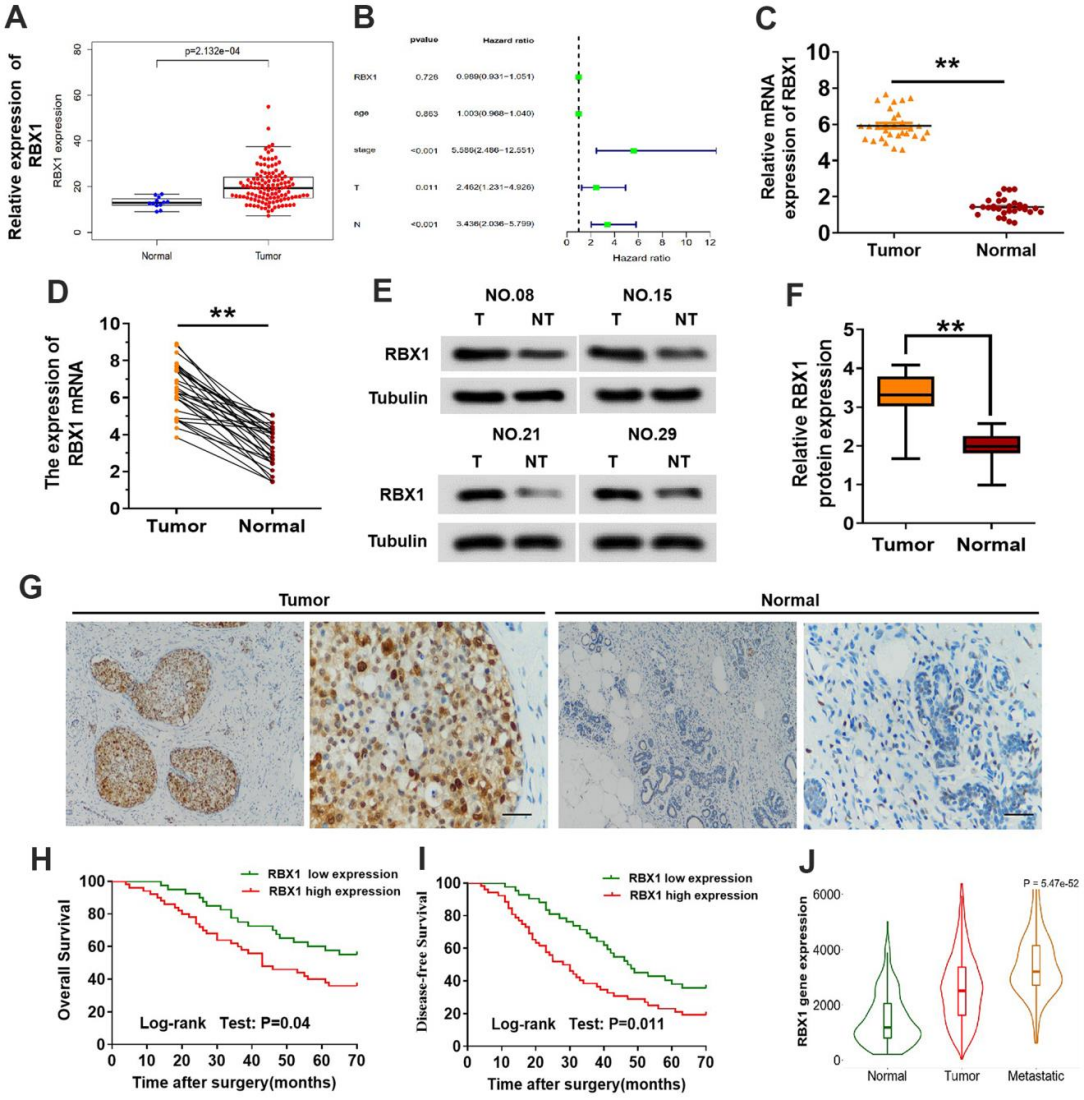
**The expression of RBX1 was over-expressed in the metastatic TNBC and greatly related to poor outcomes of patients.** (**A**) Expression of RBX1 mRNA in the dataset of TCGA TNBC cancer. (**B**) Correlation with the clinicopathological features and expression of RBX1 in patients with TNBC in TCGA cohort. (**C**, **D**) qRT-PCR was utilizing for analysing RBX1 mRNA expression levels in the tissues of TNBC tissues as well as their adjoining non-carcinoma normal tissues. ^*^P<0.05, ^**^P<0.01. (**E**, **F**) Western blotting was applying for studying RBX1 protein expression levels in the tissues of TNBC tissues together with their adjoining non-carcinoma normal tissues. ^**^P<0.01. (**G**) Immunohistochemistry was employed to identify The RBX1 protein expression existing in the tissues of TNBC and their neighboring non-carcinoma normal tissues. Scale bar, 50 μm. (**H**, **I**) For the overall survival (**H**) and the disease-free survival (**I**) in two groups of patients with TNBC, their Kaplan-Meier curves were defined by the low and high RBX1 expression, respectively. (**J**) In analysing the database of UALCAN, the expression of RBX1 was markedly elevated in metastatic and breast cancer tissues.

The underlying role of RBX1 in the TNBC development and progression was evaluated. The analysis of TCGA data suggested that RBX1 expression was raised in TNBC and that high RBX1 expression in patients with TNBC was related to distant invasion positively (Figure 1A, 1B). Subsequently, qRT-PCR was employed for determining RBX1 expression in 30 TNBC patients together with the non-tumor adjacent tissues. And the results of qRT-PCR indicated that in comparison with adjacent control tissues, RBX1 possesses a high expression in the TNBC tissues (Figure 1C, 1D). Besides, we analysed the protein levels in thirty fresh tissues of TNBC as well as their paired non-tumor adjacent tissues. In accordance with the raised RBX1 mRNA level, the RBX1 protein level was evidently higher in the TNBC tissues in contrast to non-tumor adjacent tissues (Figure 1E, 1F). The RBX1 protein levels in non-tumor adjacent tissues together with TNBC tissue samples were examined through immunohistochemical approach. In the samples in TNBC tissue, the RBX1 expression is high (Figure 1G). These findings reveal that high RBX1 levels are present in TNBC.

IHC was conducted for examining the clinicopathological features and expression of RBX1 in the TNBC specimens in order to investigate the association between clinicopathological parameters of TNBC and the overexpression of RBX1. The overexpression of RBX1 was evidently associated with the lymph node metastasis together with tumor stage (Table 1). Moreover, for exploring the influence of RBX1 on survival of patients with TNBC, the association between survival of patients with TNBC and RBX1 levels was also explored. In comparison with patients with low RBX1 levels, those with high RBX1 levels revealed lower disease-free and overall survival (Figure 1H, 1I). Further, we study the role of RBX1 in metastatic breast cancer in UALCAN database, the findings displayed that in contrast to breast cancer tissues, metastatic breast cancer has an evidently high expression of RBX1 (Figure 1J). In meantime, these data also indicate that the RBX1 is increased in the tissues of TNBC and is associated with the poor outcomes of TNBC patients.

**Table 1:** Relationship between RBX1 expression and clinicopathological features.

**Parameters**	**n**	**RBX1 expression**		**P value**
		**Low(n=73)**	**High(n=57)**	
**Age (years)**				*P*=0.327
≤60	102	55 (42.3%)	47 (36.1%)	
>60	28	18 (13.8%)	10 (7.7%)	
**Tumor size(cm)**				*P=0.392*
<2cm	44	27 (20.7%)	17 (13.1%)	
≥2cm	86	46 (35.3%)	40 (30.8%)	
**TNM**				***P=0.007***
T1-T2	43	17 (13.1%)	26 (20%)	
T3-T4	87	56 (43.1%)	31 (23.8%)	
**Distant metastasis**				***P=0.002***
No	63	44 (33.8%)	19 (14.6%)	
YES	67	29 (22.3%)	38 (29.2%)	
**Stage**				***P=0.032***
I-II	57	26 (20%)	31 (23.8%)	
III-IV	73	47 (36.2%)	26 (20%)	
**Differentiation**				*P=0.279*
Well	64	39 (30%)	25 (19.2%)	
Moderate/Poor	66	34 (26.2%)	32 (24.6%)	

### RBX1 promotes the metastasis of TNBC cells *in vitro* and *in vivo*

**Figure 2:**
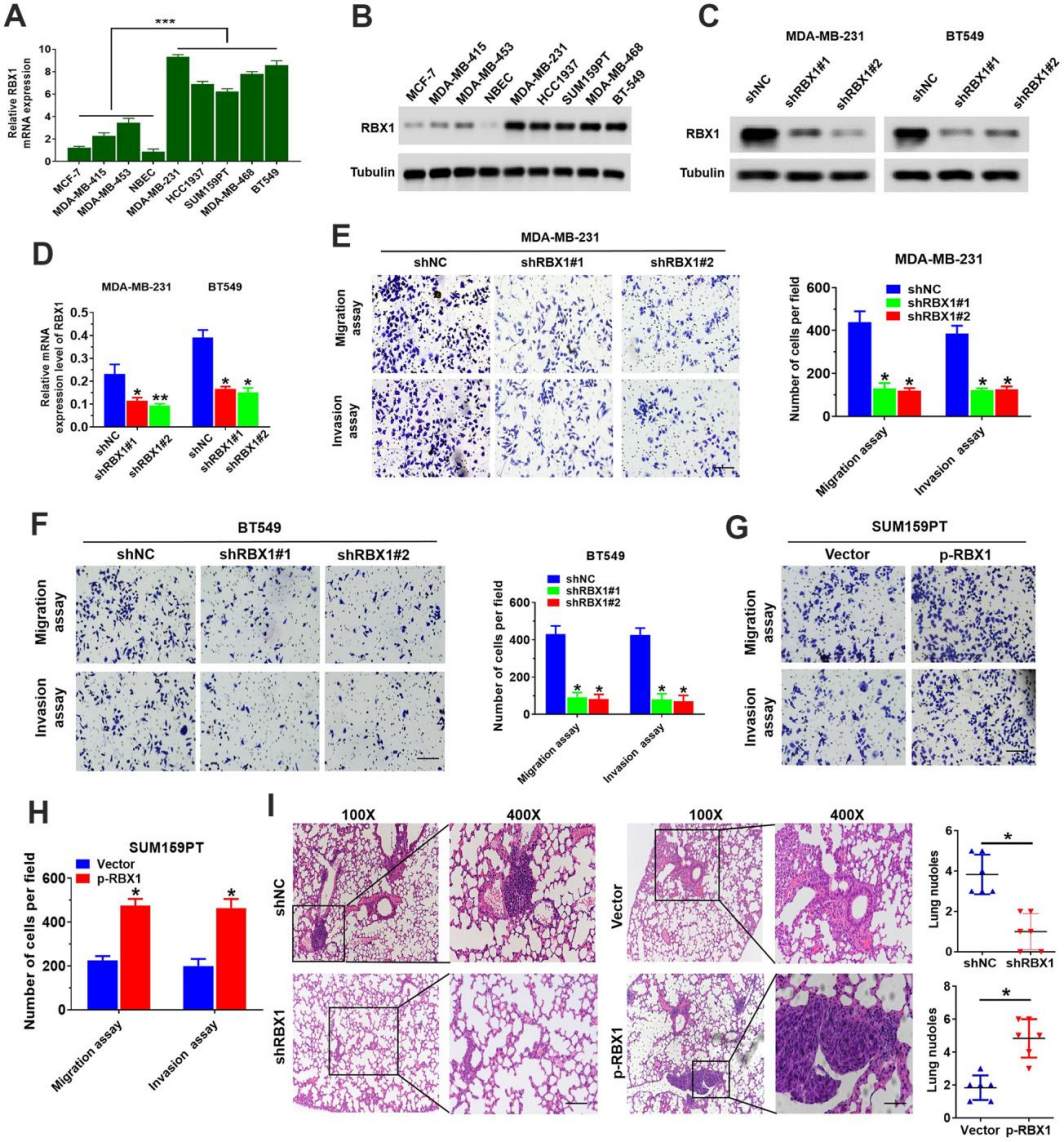
**RBX1 facilitated the cell invasion and migration of TNBC *in vivo* and *in vitro*.** (**A**, **B**) RBX1 mRNA and protein levels in normal breast epithelial, TNBC, and non-TNBC cell lines. ^***^P<0.001. (**C**, **D**) The analysis of qRT-PCR and western blot were applied for determining the RBX1 expression levels of in BT549 and MDA-MB-231 cells stably transfected with the plasmid silenced by RBX1. ^*^P < 0.05, ^**^P < 0.01. (**E**, **F**) The assays of Transwell invasion and migration of BT549 and MDA-MB-231 cells transfected by the plasmid with knockdown of RBX1. ^*^P < 0.05, ^**^P < 0.01. (**G**, **H**) The assays of Transwell invasion and migration of SUM159PT cells transfected by plasmid with overexpression of RBX1. ^*^P < 0.05, ^**^P < 0.01. (**I**) H&E staining of sections embedded with paraffin of metastatic nodules in the lungs. ^*^P < 0.05.

For the sake of exploring RBX1 expression in the BC cells, Western blotting and qRT-PCR was applied for determining the levels of its expression in five TNBC cell lines, three non-TNBC cell lines, as well as one normal breast epithelial cell line. Presence and immunoblotting data showed the RBX1 expression in five cell lines of TNBC (namely, BT549, MDA-MB-468, SUM159PT, MDA-MB-231 and HCC1937) (Figure 2A, 2B). According to the expression level of RBX1 TNBC cell line, we transfected RBX1-specific short hairpin RNA (shRBX1 # 1 and shRBX1 # 2) BT549 and MDA-MB-231 cells stably. The outcomes of Western blotting together with qRT-PCR reflected that the RBX1 expression was markedly attenuated in shRBX1 group (Figure 2C, 2D). Besides, in shRBX1 group, there is a significant reduction in the cell migration (Figure 2E, 2F). Furthermore, through matrix-coated Transwell chamber, it can be observed that compared with control group, stable RBX1 knockdown BT549 and MDA-MB-231 cells passed through matrix more slowly slower (Figure 2E, 2F). Conversely, for the TNBC cells in RBX1 overexpression group, their invasion and migration ability was significantly enhanced (Supplementary Figure 1A and Figure 2G, 2H).

The model of metastatic tumour was established by tail vein injection in nude mice, we further explored the influence of RBX1 against TNBC metastasis. And the experiment was classified as shRBX1 and shNC group. According to the histological analysis, H&E-stained serial lung sections displayed an evident reduction in the TNBC pulmonary micrometastases number in shRBX1 group. Conversely, the overexpression of RBX1 raised the pulmonary metastatic nodules number (Figure 2I). In conclusion, these outcomes exhibited that RBX1 can facilitate TNBC cells’ metastasis and invasion *in vitro* and *vivo*, reflecting that RBX1 is probably an oncogene in TNBC.

### The EMT is crucial for the oncogenic functions of RBX1

**Figure 3:**
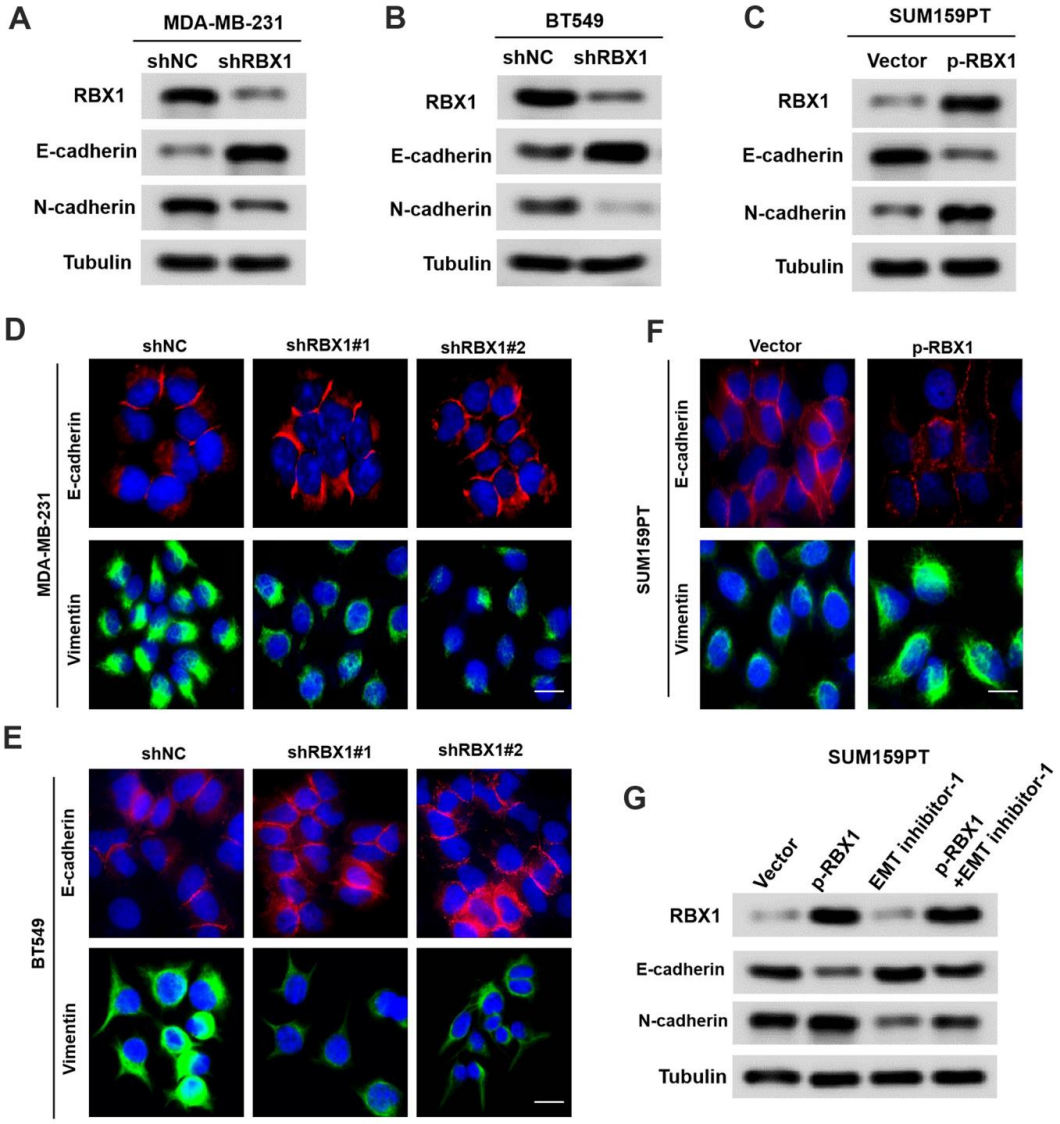
**RBX1 exerts its oncogenic role by enhancing EMT in TNBC.** (**A**, **B**) Western blot assay of the phenotypic markers of stable RBX1 silencing, containing N-cadherin and E-cadherin, in BT549 and MDA-MB-231 cells. Expression of tubulin was applied as a loading control. (**C**, **D**, **F**) Confocal microscopy assay of vimentin and E-cadherin in TNBC cells with the overexpression or knockdown of RBX1. Green and red signals reveal staining for the associated proteins, blue signal denotes the nuclear DNA staining for DAPI. (**E**) Western blot assay of the phenotypic markers, containing N-cadherin and E-cadherin in SUM159PT cells transfected with plasmid with the overexpression of RBX1. (**G**) Western blot assay of the phenotypic markers, containing N-cadherin and E-cadherin.

Up to now, EMT has been largely related to the cancer cell ability to metastasise [18]. In accordance with the influence of RBX1 on cell invasion and migration, it can be discovered that after RBX1 silencing, the expression of E-cadherin was markedly raised, while the expression of N-cadherin was attenuated in the RBX1 knockdown TNBC cells (Figure 3A, 3B). Based on the immunofluorescence analysis, epithelial markers were raised in BT549 and MDA-MB-231 cells but mesenchymal markers were reduced after RBX1 silencing (Figure 3C, 3D). Besides, according to Figure 3E, there is a remarkable increase in the expression of N-cadherin and a reduction in the expression of E-cadherin in overexpressed RBX1 cells. Additionally, with immunofluorescence analysis, it is demonstrated that in TNBC cells, the increase of RBX1 raised epithelial markers but attenuated the mesenchymal markers (Figure 3F).

To identify whether the oncogenic effect of RBX1 is determined by EMT in TNBC, EMT inhibitor-1 (C12H12Cl2N2O2S, 5mM for 24 h Med Chem Express) were used to eliminate EMT in TNBC cells transfected with vectors containing RBX1. Based on Figure 3G, the overexpression of RBX1 increased TNBC cell invasion and migration, which were eliminated by EMT inhibitors. In conclusion, these findings indicate that RBX1 exerts its carcinogenic effect by enhancing EMT in TNBC.

### RBX1 positively regulates TWIST1 protein levels

**Figure 4:**
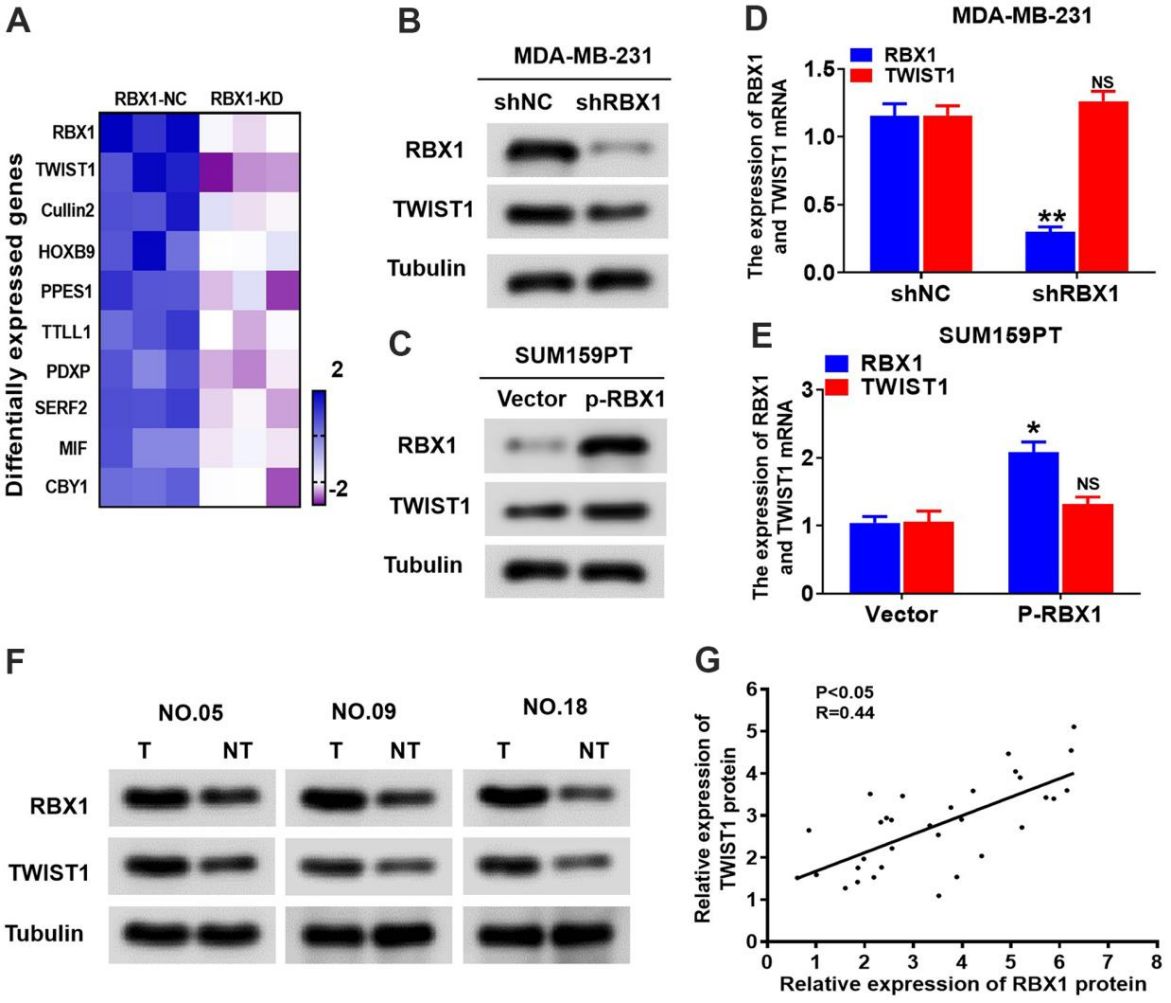
**RBX1 positively regulates the TWIST1 protein levels.** (**A**) Heatmap confirms the genes with 2-fold or more change in protein levels between the MDA-MB-231 cells with knockdown of RBX1. (**B**, **D**) The qRT-PCR and western blotting assay of the expression levels of TWIST1 in MDA-MB-231 cells stably transfected by shRBX1 or shNC plasmids. **P < 0.01. (**C**, **E**) The qRT-PCR and western blotting assay of the expression levels of TWIST1 in SUM159PT cells stably transfected by vector or plasmid with overexpression of RBX1. *P < 0.05. (**F**) Western blotting was applied for detecting the levels of TWIST1 and RBX1 protein in TNBC cancer tissues together with paired non-tumour tissues, 30 numbers in each group. Tubulin was utilized as a loading control. (**G**) Scatter plots of TWIST1 and RBX1 protein expression in the TNBC.

Previous researches have displayed that TWIST1 is a significant transcription factor that exerts a critical effect in EMT and tumour metastasis [23]. Tandem Mass Tags (TMT)-Mass Spectrometry Proteomics data reflected that the expression of TWIST1 was decreased in RBX1 knockdown TNBC cells (Figure 4A). For further identifying whether RBX1 could modulate the expression of TWIST1, we first looked at the mRNA and protein levels of TWIST1 in overexpressed or RBX1 knockdown TNBC cancer cells. Based on the western blot analysis, RBX1 attenuation markedly decreased TWIST1 protein expression existing in the MDA-MB-231 cells (Figure 4B). Conversely, RBX1 overexpression remarkably raised the TWIST1 protein level in the TNBC cells (Figure 4C), whereas the TWIST1 mRNA expression was not affected in RBX1 knockdown or overexpression in TNBC tumour cells (Figure 4D, 4E). Next, we determined the TWIST1 protein level in 30 RBX1 up-regulated TNBC tumour tissues using western blotting. Our findings displayed that in TNBC cancer tissues, the TWIST1 protein level was markedly increased in comparison with the level in associated adjacent normal tissues (Figure 4F). As the scatter plot reflected, between the TWIST1 and RBX1 expression levels in TNBC, there existed a positive association (Figure 4G).

### TWIST1 is key for RBX1 mediated TNBC invasion and metastasis *in vitro* and *in vivo*

**Figure 5:**
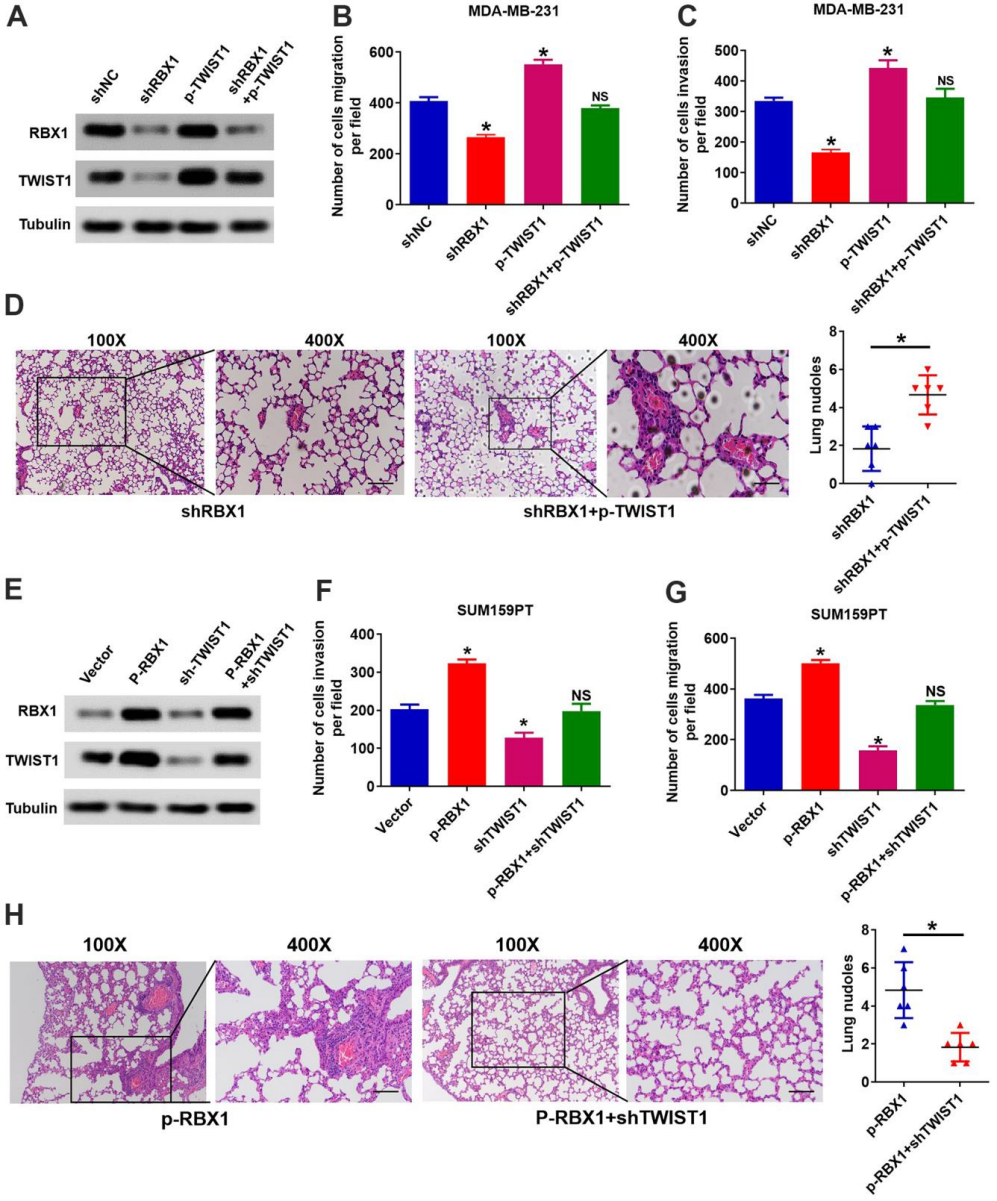
**RBX1 facilitates TNBC metastasis and invasion via raising the expression of TWIST1.** (**A**) The expression of TWIST1 and RBX1 was measured with western blotting. The raise of TWIST1 reduced the expression of TWIST1 in MDA-MB-231-shRBX1 cells. (**B**, **C**) Transwell analysis suggested that the increase of TWIST1 markedly rescued the cell invasion and migration in the MDA-MB-231-shRBX1 cells (*P<0.05). (**D**) H&E staining of sections embedded with paraffin of metastatic nodules in the lung. *P < 0.05. (**E**) Western blotting was applied for measuring the levels of TWIST1 and RBX1 protein. The TWIST1 expression knockdown greatly suppressed the raise in the expression of TWIST1 in SUM159PT-p-RBX1 cells. (**F**, **G**) Transwell analysis displayed that TWIST1 suppression attenuated RBX1-enhanced cell invasion and migration. *P < 0.05. (**H**) H&E staining of sections embedded with paraffin of metastatic nodules in the lung. * *P* < 0.05.

We further validated that the RBX1 mediates metastasis and invasion of TNBC through regulation of TWIST1. We raised the TWIST1 expression in the RBX1 knockdown TNBC cells, and subsequently, the Transwell and Western blot analysis was applied for observing the TWIST1 and RBX1 proteins expression levels together with the cell invasion and migration ability. According to Figure 5A, the analysis of Western blot exhibited that the increase of TWIST1 reduced the TWIST1 expression loss in the RBX1 knockdown MDA-MB-231 cells. It can also be observed that TWIST1 increase reversed the ability of invasion and migration resulted from RBX1 knockdown (Figure 5B, 5C). Furthermore, *in vivo* metastasis assays indicated that TWIST1 upregulation could raise the incidence of lung metastasis existing in MDA-MB-231-shRBX1 group (Figure 5D).

Afterward, we decrease the TWIST1 expression in RBX1 overexpressed TNBC cells, and observed the protein levels of RBX1 and TWIST1 as well as the ability of cells to invade and migrate. The outcomes of Western blot displayed that the expression of TWIST1 can be raised evidently by the RBX1 overexpression, but the reduced expression of TWIST1 markedly suppressed the increase in the expression of TWIST1 resulted from RBX1 in SUM159PT cells (Figure 5E). Besides, the decrease of TWIST1 remarkably attenuated RBX1 and raised the invasion and migration of cell (Figure 5F, 5G). Thus, the outcomes of the *in vivo* metastasis assay suggested that attenuation of TWIST1 reduced the incidence of lung metastasis existing in SUM159PT-p-RBX1 group (Figure 5H). As a result, these findings demonstrated that TWIST1 is critical for the metastasis and invasion of TNBC mediated by RBX1.

### RBX1 mediates ubiquitination and degradation of FBXO45

**Figure 6:**
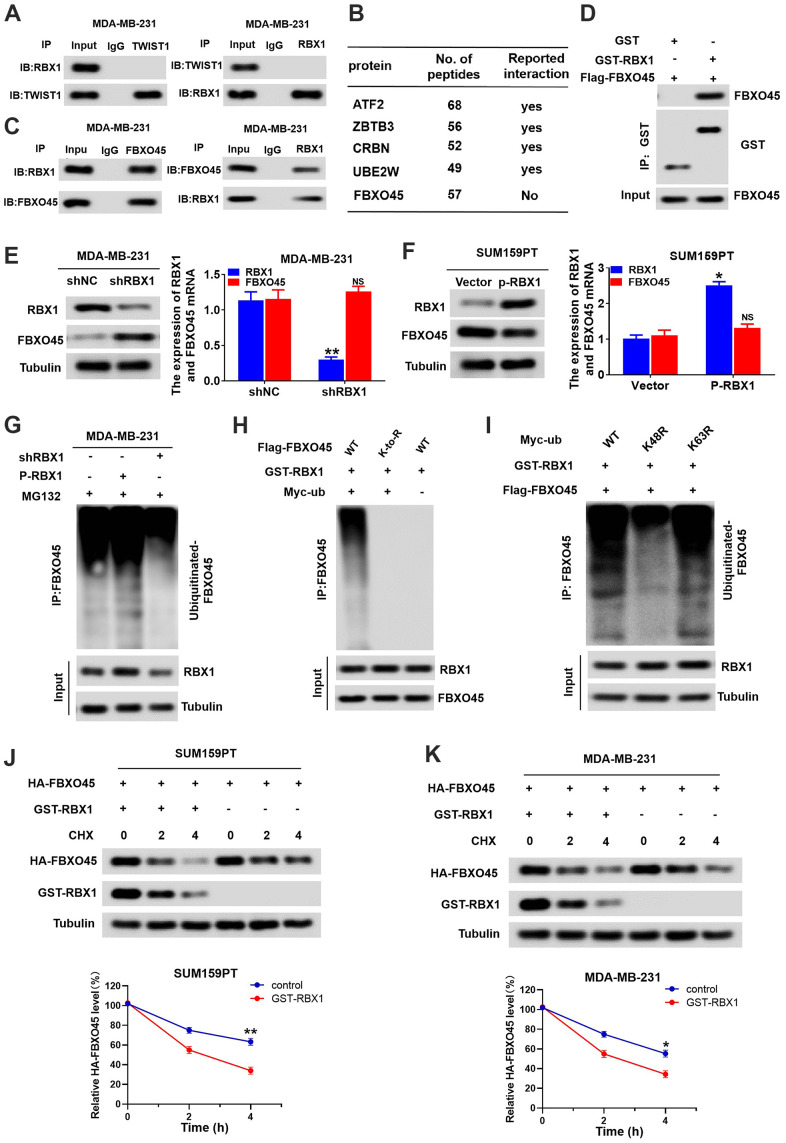
**RBX1 promotes FBXO45 ubiquitination and degradation in TNBC cells.** (**A**) Co-IP reveals no direct binding of endogenous TWIST1and RBX1. (**B**) Partial list of proteins related to RBX1, determined with immunoprecipitation-mass spectrometry. (**C**) Co-IP reflected direct binding of endogenous FBXO45 and RBX1 in the MDA-MB-231 cells. (**D**) GST pull-down analysis displaying direct binding of FBXO45 and RBX1. (**E**) qRT-PCR and western blotting exhibiting the FBXO45 and RBX1 expression levels in the RBX1-silencing MDA-MB-231 cells. **P < 0.01. (**F**) The qRT-PCR and western blotting revealing the FBXO45 and RBX1 expression levels in SUM159PT cells stably transfected by plasmid with overexpression of RBX1. **P < 0.01. (**G**) RBX1 exogenous expression or knockdown changed the FBXO45 ubiquitination. The proteasome suppressor MG132 was used to treat the cells from each group. Cell lysates were generated and next immunoprecipitated by an anti-FBXO45 antibody. Western blotting was applied for measuring the levels of ubiquitin-linked FBBXO45 utilizing anti-UB antibody. (**H**) Ubiquitination of wild-type FBBXO45 or K-to-R mutants (mutations in all Lys position of the FBBXO45 gene). (**I**) FBXO45 ubiquitination in the HEK293 cells was detected. (**J**, **K**) TNBC cells were transfected by plasmids encoding HA-FBXO45, without or with p-RBX1 plasmid. Cells were next exposed to 20μmol/L of cyclohexanone (CHX) at the given times, and the anti-HA antibody was utilized to determine FBXO45 degradation. *P < 0.05, **P < 0.01.

With an aim of further clarifying the mechanism that RBX1 modulates TWIST1 in the TNBC cells, we first investigated whether there existed a direct interaction between TWIST1 and RBX1 proteins. It has been reported that RBX1 interacts with various substrates to exert its function. Nevertheless, the co-IP revealed that there existed no direct interaction between TWIST1 and RBX1 proteins. (Figure 6A). The above outcomes reflected that RBX1 modulates the TWIST1 expression positively in TNBC cells. RBX1, as an E3 ubiquitin ligase, is responsible for protein recycling and degradation. Next, we assumed that RBX1 may raise the TWIST1 expression level by degrading the expression of TWIST1 negative regulatory genes. To determine how RBX1 regulates TWIST1 expression, we identified RBX1 interacting proteins in TNBC cells using mass spectrometry. Through the mass spectrometry, we determined a total of 128 proteins, and observed that only FBXO45 (a significant cellular antagonist of TWIST1) may interact with RBX1 (Figure 6B). In addition, FBXO45 has been shown to be associated with the progression of various tumours. Further, co-IP detection data confirmed the interaction between RBX1 and FBXO45 in TNBC cells (Figure 6C). Moreover, the GST pull-down tests exhibit that RBX1 can bind with FBXO45 under the cell-free environments (Figure 6D). According to these results, RBX1 interacts directly with FBXO45 in TNBC cells.

To in-depth identify whether the RBX1 can modulate the FBXO45 expression, the FBXO45 mRNA and protein levels in overexpressed or RBX1 knockdown TNBC cells were observed first. Based on Figure 6E, qRT-PCR and western blotting outcomes reflected that FBXO45 protein levels were remarkably raised in MDA-MB-231 cells after the knockdown of RBX1. Conversely, the RBX1 overexpression evidently attenuated the FBXO45 protein level existing in the SUM159PT cells (Figure 6F). FBXO45 mRNA expression was not influenced in the overexpressed or RBX1 knockdown TNBC cells (Figure 6E, 6F). As a result, these outcomes reflected that RBX1 can attenuate the FBXO45 expression. Next, we investigated whether RBX1 directly mediates FBXO45 ubiquitination. Interestingly, ectopic expression of RBX1 led to an evident raise in the polyubiquitination of FBXO45, but knockdown of RBX1 induced a reduction in the polyubiquitination of FBXO45 (Figure 6G). The data indicated that at all Lys positions of FBXO45, the mutations eliminated FBXO45 polyubiquitination (Figure 6H). As expected, on ubiquitin (UB), the lys48 mutation nearly fully eliminated the ubiquitination of FBXO45 mediated by RBX1, while k63R mutation on UB possessed no influence (Figure 6I). In accordance with the ubiquitination outcomes, degradation kinetic experiments reflected that in contrast to control cells, the exogenous FBXO45 half-life in the TNBC cancer cells overexpressing RBX1 was markedly lower (Figure 6J, 6K). These data suggest that RBX1 mediates the polyubiquitination of the lys48 chain of FBXO45, leading to the FBXO45 degradation in the proteasome.

### RBX1 accelerates the invasion and metastasis of TNBC through a FBXO45-TWIST1-dependent manner

**Figure 7:**
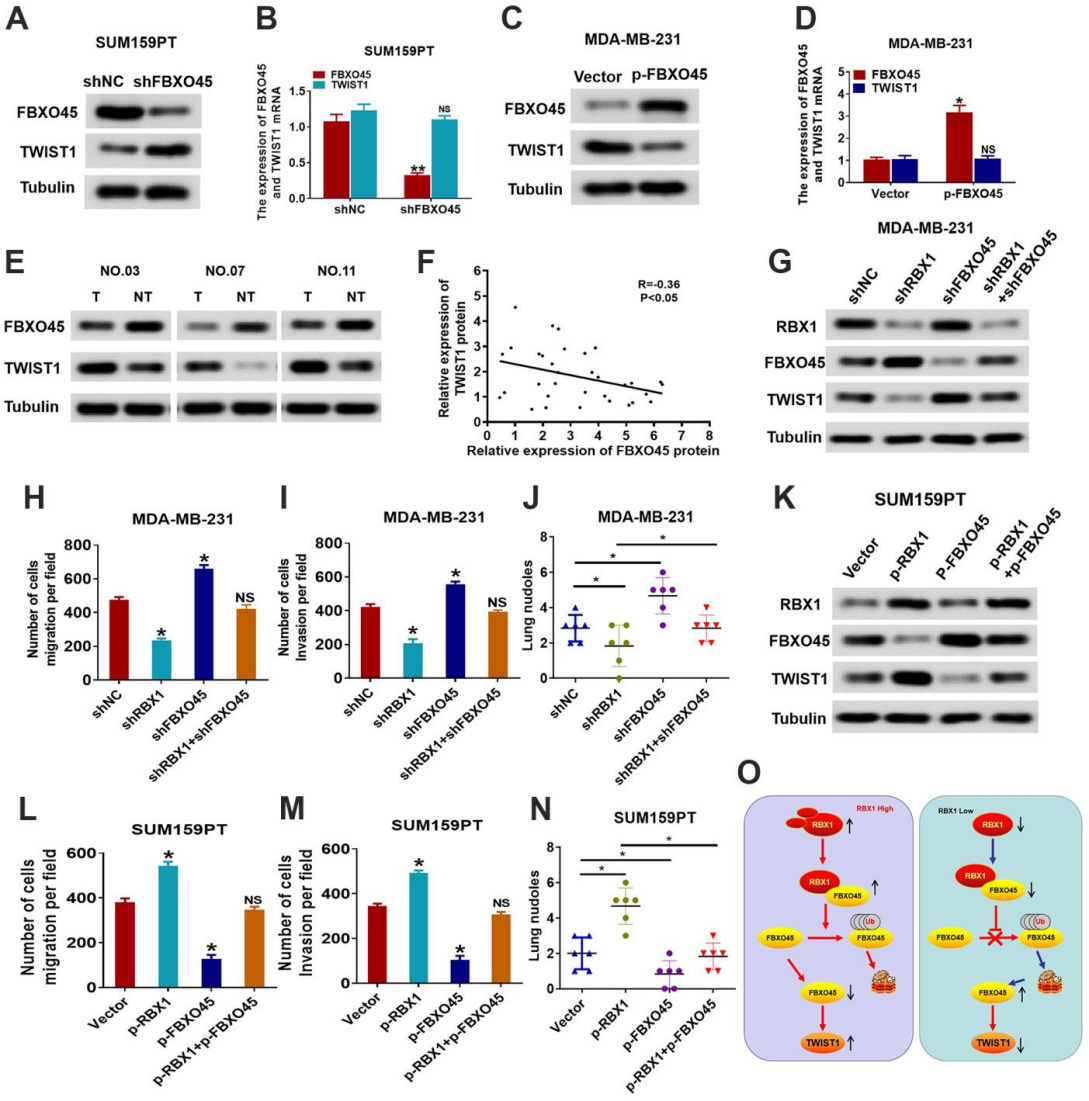
**RBX1 promotes the TWIST1-mediated migration dependent on FBXO45.** (**A**, **B**) The qRT-PCR and western blotting of the TWIST1 expression levels in the SUM159PT cells stably transfected by shFBXO45 or shNC plasmid. *P < 0.05. (**C**, **D**) The qRT-PCR and western blotting of the TWIST1 expression levels in the MDA-MB-231 cells stably transfected by vector or plasmid with the overexpression of FBXO45. *P < 0.05. (**E**) Western blotting was applied for detecting the levels of TWIST1 and FBXO45 protein in TNBC cancer tissues together with paired non-tumour tissues, 30 numbers in each group. Tubulin was utilized as a loading control. (**F**) Scatter plots of TWIST1 and FBXO45 protein expression in the TNBC. (**G**) Western blot of TWIST1, FBXO45, and RBX1 protein levels in the MDA-MB-231 cells stably transfected by shRBX1 with or without shFBXO45. The expression of tubulin was employed as a loading control. (**H**, **I**) Transwell analyses were implement to identify the MDA-MB-231 cells metastatic capacity that stably transfected by shRBX1 with or without shFBXO45. *P < 0.05. (**J**) The data reflect the statistics of the number of nodules of lung metastases. *P < 0.05. (**K**) Western blot of TWIST1, FBXO45, and RBX1 protein levels in the TNBC cells stably transfected by p-RBX1with or without p-FBXO45. The expression of tubulin was employed as a loading control. (**L**, **M**) Transwell analyses were implement to identify the TNBC cells metastatic capacity that stably transfected by p-RBX1 with or without p-FBXO45. *P < 0.05. (**N**) The data display the statistics of the number of nodules of lung metastases. *P < 0.05. (**O**) Schematic representation of E3 UB ligase RBX1 driving the transfer of TNBC via an FBXO45-TWIST1-dependent degradation mechanism.

Next, we assessed whether RBX1 affects the expression of TWIST1 through FBXO45. To this end, we first measured changes in FBXO45 and TWIST1 protein and mRNA expression in FBXO45 knockdown SUM159PT cells. The outcomes reflected that the knockdown of FBXO45 obviously raised the TWIST1 protein expression in SUM159PT cells, but had no effect on the mRNA expression of TWIST1 in SUM159PT cells (Figure 7A, 7B). Conversely, FBXO45 overexpression obviously attenuated the protein expression of TWIST1 in the MDA-MB-231 cells (Figure 7C). Nonetheless, TWIST1 mRNA levels did not influence the variations in MDA-MB-231 cells of FBXO45 overexpression (Figure 7D). Furthermore, the TWIST1 and FBXO45 protein levels were measured in thirty TNBC tissues through applying the western blotting. Our findings displayed that the FBXO45 protein levels were obviously attenuated in the TNBC cancer tissues in comparison with the associated adjoining normal tissues (Figure 7E). The scatter plot exhibited that the TWIST1 and FBXO45 protein expression levels were associated negatively in the tissues of TNBC (Figure 7F). These findings suggest that FBXO45 regulates TWIST1 expression in TNBC cells.

We then conducted a rescue experiment to determine whether RBX1 accelerates tumour invasion and metastasis in an FBXO45-TWIST1-dependent manner. First, we further inhibited the expression of FBXO45 in the MDA-MB-231 cells that interfered with RBX1, and then observed the expression level and cell migration ability of the FBXO45 and TWIST1 proteins. As reflected in Figure 7G, the outcomes of western blotting suggested that the decrease of FBXO45 markedly suppressed the reduction in the expression of TWIST1 in MDA-MB-231 cells with the knockdown of RBX1. Meanwhile, downregulation of FBXO45 decreased the metastasis and invasion resulted from the knockdown of RBX1 *in vitro* and *in vivo* (Figure 7H–7J). Conversely, *in vivo* and *in vitro*, the FBXO45 overexpression significantly attenuated the loss of FBXO45 expression and significantly reduced cell invasion and metastasis of RBX1 overexpressed SUM159PT cells (Figure 7K–7N). Thus, these results suggest that RBX1 accelerates TNBC invasion and metastasis in an FBXO45-TWIST1 dependent manner.

## DISCUSSION

Although progress has been made in the prognosis and treatment of TNBC, there has been no significant progress in raising the survival rate. Therefore, it is essential to further understand the biological features of TNBC [29]. The significance of EMT in tumourigenesis and development has been elucidated only recently. In contrast to other subtypes, the TNBC cells possess the activated EMT process, and this a highly dynamic process that can polarize excellent epithelial cells into the mesenchymal cells, containing variations in cell-matrix and cell-cell adhesion, higher metastatic potential, raised cell invasiveness and mobility, the remodelling of the cytoskeleton and loss of polarity, as well as resistance to effective therapy [30, 31]. Therefore, understanding the pathogenesis of TNBC from the perspective of EMT-MET will improve our understanding of breast cancer. The high RBX1 expression reflects that there is a poor TNBC prognosis at present. RBX1 exerts an essential effect in the EMT-MET reprogramming of TNBC.

RBX1, also called ROC1, is a major component of E3 ubiquitin ligase [8, 9]. It has evolved from yeast to humans and has exerted a major effect in the development of embryos [9, 15]. Recent researches have exhibited that RBX1 not only exerts a critical role in modulating several cellular physiological functions, but is also participated in the cancer development [8, 15]. RBX1 is overexpressed in many primary tumours, including liver cancer, non-musculoinvasive transitional cell carcinoma of the bladder (NMIBC), and lung cancer [32–36]. Several studies have shown that RBX1 is participated in different aspects of the cancer biology, and lots of RBX1 molecular functions are in accordance with its function in cancer [37–39]. Nonetheless, there exist no information on the RBX1 molecular mechanisms and its concrete role in TNBC. In the current work, our outcomes exhibited that the expression levels of RBX1 were raised in the tumours of TNBC patients in contrast to the associated non-tumour tissues. What’s more, in patients with TNBC, the overexpression of RBX1 was remarkedly related to overall survival, lymph node metastasis stage together with tumour stage. Further investigations displayed that RBX1 facilitated the metastasis and invasion of TNBC cell *in vivo* and *in vitro*. Hence, these findings exhibit that RBX1 is probably a new indicator of poor TNBC outcomes and may serve as an oncogene in TNBC progression.

For the tumour cells, their MET-EMT transformation is conducive to their distant metastasis and survival in the hostile microenvironment [40, 41]. Hence, further understanding the underlying MET-EMT transformation regulatory mechanisms in TNBC cells may be conducive to determine novel therapeutic opportunities for patients with TNBC [42]. Recent researches have emphasised the significance of MET-EMT transformation in the TNBC development. For example, Yoshida et al. demonstrated that methanesulfonic keratin inhibits the experimental metastasis of breast cancer cells by reversing the phenotype from EMT to MET [42]. Here, we confirmed that RBX1 is closely related to MET-EMT transformation in TNBC cells. Our results show that RBX1 can promote the transformation of MET into EMT in TNBC cells. These outcomes are significant for in-depth understanding the RBX1 bio-function in the TNBC, simultaneously for evaluating the RBX1 potentiality as a treatment target.

In order to explore the mechanism that RBX1 influences MET-to-EMT, we emphasize TWIST1, a bHLH transcription factor that induces EMT [23]. Recently, several researches have exhibited the importance of TWIST1 in the genesis and progression of cancer [24]. Nevertheless, the effect of TWIST1 expression in the TNBC progression is still not explicit. Here, we reveal a novel mechanism that inhibits TNBC migration and EMT through the RBX1 silencing-mediated reduction of TWIST1 expression. First, it can be discovered that in the tissues of TNBC, the TWIST1 and RBX1 expression levels were high, and between these expression levels, there existed a positive association. Besides, our data also revealed that the decrease of RBX1 attenuate the TWIST1 expression and inhibited TNBC migration and EMT. In addition, TWIST1 is critical to TNBC metastasis and invasion mediated by RBX1 *in vivo* and *in vitro*. Briefly, these data reflect that RBX1 modulates the expression of TWIST1 to affect the TNBC metastasis and invasion, so as to determining new TWIST1 regulatory mechanisms.

Next, we studied the molecular mechanism of RBX1 regulating TWIST1 expression. Investigations have exhibited that FBXO45 exerts a significant effect in modulating the progression of cancer [26, 27]. This work displayed a new mechanism that RBX1 modulates the expression of TWIST1 through influencing the expression of FBXO45. Such conclusion is acquired according to the below observations. First, our outcomes exhibited that the TWIST1 and FBXO45 expression levels were negatively associated in TNBC tumour tissues, and FBXO45 regulated TWIST1 expression in TNBC cells. Second, downregulation of FBXO45 significantly inhibited the decrease in the expression of TWIST1 in the MDA-MB-231 cells with knockdown of RBX1. In meantime, the downregulation of FBXO45 blocked invasion and metastasis caused by RBX1 gene knockdown *in vivo* and *in vitro*. In conclusion, such data exhibit that RBX1 accelerates the transfer of TNBC and EMT in an FBXO45-TWIST1 dependent manner.

Finally, we studied the mechanism by which RBX1 regulates FBXO45. The FBXO45 degradation mediated with UB-protease is a critical mechanism modulating intracellular FBXO45 levels. In accordance these data, our findings indicate firstly that RBX1 is participated in the FBXO45 degradation and that it may utilize as the FBXO45 E3 ubiquitin ligase. This summary is obtained these observations. First, data from co-IP and GST pull-down analyses demonstrated the interaction between FBXO45 and RBX1 in TNBC cells. Second, the knockdown of RBX1 remarkably raised the FBXO45 protein level, but the expression of FBXO45 mRNA was not affected in TNBC cancer cells with RBX1 knockdown. Third, we also found that the polyubiquitination of FBXO45 is raised and attenuated with the ectopic expression of RBX1 and the knockdown of RBX1, respectively, and mutations at all Lys sites of FBXO45 cancelled FBXO45 polyubiquitination. In addition, degradation kinetics analysis exhibited that for the exogenous expression of FBXO45, its half-life was obviously raised in the TNBC with RBX1 overexpression.

To sum up, we give the first evidence that in TNBC, RBX1 is raised and it is related to disease progression in TNBC patients. We also discovered that RBX1 promoted TNBC cell migration and EMT. What’s more, EMT induced by RBX1 is determined by the TWIST1 expression in TNBC cells. Our outcomes also display that RBX1 modulates the TWIST1 expression through modulating FBXO45, which is accomplished through binding to FBXO45 directly and facilitating its degradation and ubiquitination (Figure 7O). In accordance with these outcomes, RBX1 could be a candidate biomarker for the future TNBC diagnosis together with its treatment.

## MATERIALS AND METHODS

### Cells and tissues

Normal breast epithelial cells (NBECs) were cultivated following the instructions of manufacturer. From ATCC, the breast cancer cell lines could be acquired, including MDA-MB-453, MCF-7, MDA-MB-415, BT-549, MDA-MB-468, SUM159PT, MDA-MB-231 and HCC1937, which were stored in the DMEM medium routinely (Invitrogen, Carlsbad, CA, USA) added with foetal bovine serum (10%, HyClone, Logan, UT, USA). While Second Affiliated Hospital of Nanchang University provided the breast cancer tissues. And the normal breast tissues were provided by the person who had died as a result of a traffic accident and were demonstrated to be free of any pre-presenting pathological test situations. The donor consent together with Institutional Research Ethics Committee approval were acquired.

### *In vivo* metastasis assay

The influence of RBX1 against BC metastasis was analysed in lung metastasis model. Each group has 6 female BALB/C nude mice (from 4 to 6 weeks; between 18 and 20g; Shanghai SLAC Experimental Animal Co., Ltd.), and the experimental environment adopted the barrier system standardised by the experimental animal centre of Nanchang University. Each nude mouse was injected in the tail vein for 1× 10^6^ MDA-MB-231 cells in 100μl PBS. Six weeks after the implantation of tumour, the mice were sacrificed. And the nude mice were euthanized with the cervical dislocation. Euthanasia was determined by observing respiration, the corneal reflex, and heartbeat. The criteria for animal mortality were lack of breathing, heartbeat, and corneal reflex. The lungs and the main organs were also observed. Subsequently, the organs were fixed, embedded, and sectioned. The metastasis was determining through eosin and hematoxylin staining. The experiments of animal were authorized through the Animal Experiment Ethics Committee of the Second Affiliated Hospital of Nanchang University.

### Migration and invasion assay

The invasion and migration assays based on Transwell in cell lines of TNBC were implemented as previously mentioned, with slight changes. In the upper part of test chamber, the polycarbonate membrane was pre-coated by a matrix gel in order to conduct the assay of cell invasion.

### Co-immunoprecipitation (Co-IP) and glutathione S-transferase (GST) pull-down experiments

For carrying out co-IP analysis, BC cells with the overexpression or knockdown of RBX1 were exposed to MG132 for four hours prior to harvest. Afterward, anti-FBXO45 antibody was applied for immunoprecipitating the cell lysates and anti-UB antibody was utilized for detecting the FBXO45 UB levels. The BL-21-active *E. coli* (Beijing Solarbio, China) were transformed through the indicated plasmids and next incubated overnight at RT for GST pull-down analysis. Isopropyl β-d-thiogalactopyranoside leads to the expression of GST fusion proteins. Cells were collected in lysis buffer and homogenised using ultrasound. Based on the instructions of manufacturer, glutathione Sepharose 4 B beads (GE Healthcare) was used for purifying GST fusion protein from the supernatant after centrifugation. Equal fractions of GST-RBX1 protein or glutathione (5 μg) were added into the lysate of breast cancer cell, which were then gently rotated overnight at room temperature and added with glutathione Sepharose 4 B beads (GE Healthcare) for three hours. The beads could be gathered with centrifugation and next washed through utilizing cold cracking buffer. Western blotting was employed for the determination of FBXO45 protein.

### Cycloheximide (CHX) chase assay

CHX (20μmol/L, Sigma Aldrich; Merck) was applied to treat the cells for the sake of suppressing the generation of FBXO45 protein in the BC cells. Lipofectamine 3000 (Invitrogen; Thermo Fisher Scientific, Waltham, MA, USA). After transfection for 2 days, cells were gathered at various time points (0, 2 and 4h) after the treatment of CHX. The FBXO45 protein expression was tested through Western blotting.

### Statistical analysis

The data are expressed as the mean of three independent tests. Student’s t-test as well as the Dunnett’s post-hoc and one-way ANOVA test were utilized for analysing the differences between two groups and between multiple groups respectively. The correlation between the clinicopathological characteristics and expression of RBX1 of patients with BC was analysed with Fisher’s exact probability test. While utilizing Tukey’s post-hoc test and one-way ANOVA test, multiple comparisons could be implemented. Differences were regarded as statistically significant when *P* < 0.05. SPSS 19.0 (SPSS Company) and GraphPad Prism 6.0 (GraphPad Software Company, San Diego, CA, USA) were applied for implementing all the statistical calculations and plotting all charts, respectively. When *P* < 0.05, differences were viewed as statistically significant.

## Supplementary Material

Supplementary Figure 1
