# Supplementary material for: E3 ubiquitin ligase RBX1 drives the metastasis of triple negative breast cancer through a FBXO45-TWIST1-dependent degradation mechanism
Source: Aging (Albany NY). 2022 Jul 8;14(13):5493–510. doi: 10.18632/aging.204163 (PMC9320552; doi:10.18632/aging.204163)
Supplement: Supplementary Figure 1 [file aging-14-204163-s001.pdf]

SUPPLEMENTARY FIGURE

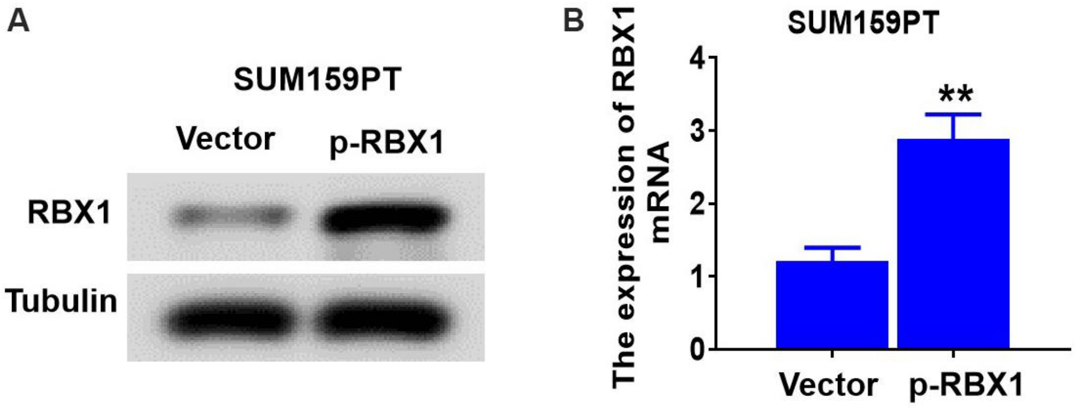

Supplementary Figure 1. Overexpress RBX1 in SUM159PT cells. (**A**, **B**) Western blot and qRT-peR analyses were used to detect the expression levels of RBX1 in SUM159PT cells stably transfected with the p-RBX1 plasmid. *P < 0.05, **P < 0.01.
